# Non-active implantable device treating acid reflux with a new dynamic treatment approach: 1-year results: RefluxStop™ device; a new method in acid reflux surgery obtaining CE mark

**DOI:** 10.1186/s12893-020-00794-9

**Published:** 2020-07-20

**Authors:** Miloš Bjelović, László Harsányi, Áron Altorjay, Zsolt Kincses, Peter Forsell, Investigators of the RefluxStop™ Clinical Investigation Study Group, Dragan Gunjić, Milan Veselinović, Tamara Babič, Péter Lukovich, Timea Kakucs, Sándor Kathy

**Affiliations:** 1 Department for Minimally Invasive Upper Digestive Surgery, University Hospital for Digestive Surgery - First Surgical Hospital, Clinical Center of Serbia; University of Belgrade, School of Medicine, Belgrade, Serbia; 2 1st Department of Surgery, Semmelweis University, Budapest, Hungary; 3 Surgical Department, Fejér County Szent György University Teaching Hospital, Székesfehérvár, Hungary; 4 General Surgery Department, University of Debrecen Kenézy Gyula Teaching Hospital, Debrecen, Hungary; 5 Inventor of RefluxStop™, Seehof 4b, 6072 Sachseln, Switzerland

**Keywords:** Gastroesophageal reflux disease (GERD), Treatment, Surgery, RefluxStop™, Dysphagia, pH monitoring, GERD-HRQL, Gas bloating

## Abstract

**Background:**

RefluxStop™ is an implantable, non-active, single use device used in the laparoscopic treatment of GERD. RefluxStop™ aims to block the movement of the LES up into the thorax and keep the angle of His in its original, anatomically correct position. This new device restores normal anatomy, leaving the food passageway unaffected.

**Methods:**

In a prospective, single arm, multicentric clinical investigation analyzing safety and effectiveness of the RefluxStop™ device to treat GERD, 50 subjects with chronic GERD were operated using a standardized surgical technique between December 2016 and September 2017. They were followed up for 1 year (CE-mark investigation 6-months). Primary safety outcome was prevalence of serious adverse events related to the device, and primary effectiveness outcome reduction of GERD symptoms based on GERD-HRQL score. Secondary outcomes were prevalence of adverse events other than serious adverse events, reduction of total acid exposure time in 24-h pH monitoring, and reduction in average daily PPI usage and subject satisfaction.

**Results:**

There were no serious adverse events related to the device. Average GERD-HRQL total score at 1 year improved 86% from baseline (*p* < 0.001). 24-h pH monitoring compared to baseline showed a mean reduction percentage of overall time with pH < 4 from 16.35 to 0.80% at the 6-month visit (*p* < 0.001), with 98% of subjects showing normal 24-h pH. At 1 year: No new cases of dysphagia were recorded, present in 2 subjects, which existed already at baseline. Regular daily PPI usage occurred in all 50 subjects at baseline. At 1-year follow-up, only 1 subject took regular daily PPIs due to a too low placement of the device thereby prohibiting its function. None or minimal occasional episodes of regurgitation occurred in 97.8% of evaluable subjects. Gas bloating disappeared in 30 subjects and improved in 7 subjects.

**Conclusion:**

The new principle of RefluxStop™ is safe and effective to treat GERD according to investigation results. At 1-year follow-up, both the GERD-HRQL score and 24-h pH monitoring results indicate success for the new treatment principle. In addition, with the dynamic treatment for acid reflux, which avoids compressing the food passageway, prevalence of dysphagia and gas bloating are significantly reduced.

**Trial registration:**

ClinicalTrials.gov, NCT02759094. Registered 3 May, 2016,

## Background

Anti-reflux surgery has been focused on supporting the Lower Esophageal Sphincter (LES) for the past 65 years. Nissen fundoplication [1–22], as one standard of care, compresses the food passageway causing dysphagia, odynophagia and inability to belch or vomit as well as associated gas bloating. The long-term result is also plagued by a 36% failure rate when including 25% PPI users and an 11% reoperation rate [23]. This is likely caused by the fact that the cuff becomes thinner and more inelastic over time due to inactivation of the stomach wall.

**Fig. 1:**
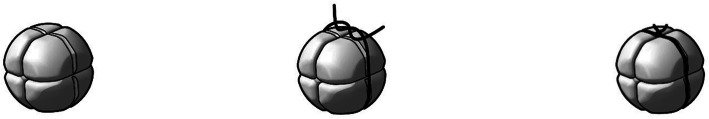
RefluxStop™ implant. RefluxStop™ implant - illustration without suture, followed by assembly and suture placement

RefluxStop™ is an implantable, non-active, single use sterile device to be used in the laparoscopic treatment of GERD (Fig. 1).

The thesis behind RefluxStop™ is that acid reflux is caused by two malfunctioning events, both of which are addressed with this new device. First, the belching process with fundus contraction and simultaneous relaxation of the LES also includes fluid due to the anatomical misalignment of the angle of His. Furthermore, acid reflux is caused by the lower esophageal sphincter temporarily or permanently entering into the chest. The pressure in the abdomen supports the LES to close while when in the chest, due to the abnormal thorax position with weaker pressure support combined with the breathing process, the closing function is often not working properly resulting in acid reflux. During the breathing process, the diaphragm moves up and down. This fact has been underestimated as the cause of acid reflux and a new dynamic anti reflux treatment is needed.

RefluxStop™ reinforces the fundus to interact with the diaphragm for a dynamic treatment of acid reflux. It is placed on the outside of the stomach top fundus wall with laparoscopic surgery. The RefluxStop™ procedure reconstructs the angle of His and reinforces the top part of the stomach (fundus) by invagination of the device in the pocket created out of the anterior wall of the fundus. RefluxStop™ aims to block the movement of the LES up into the thorax and keep the angle of His in its original anatomically correct position. This new device restores normal anatomy (with the LES remaining in the abdomen) by dynamically acting like a mechanical stop against the diaphragm muscle parallel to the LES and the hiatus opening in the diaphragm, leaving the food passageway unaffected. Therefore, side effects associated with “gold standard” surgery are reduced when avoiding compression of the food passageway.

## Methods

### Study design and objectives

**Fig. 2:**
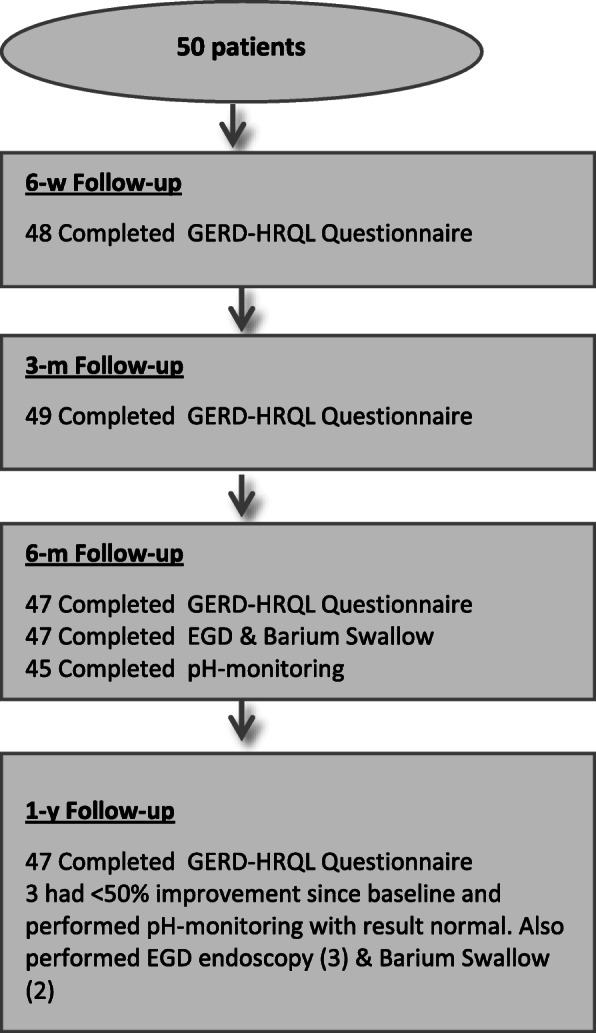
Study Schedule of Assessments: Baseline to Year 1 visit

In a prospective, single arm, multicentric CE mark clinical investigation analyzing safety and effectiveness of the RefluxStop™ device to treat GERD, 50 subjects with chronic GERD were included. Subjects were operated using a standardized surgical technique in the period between December 2016 and September 2017 and followed up for 1 year with the CE mark obtained based on 6-month follow-up. Gastroscopy, contrast swallow x-ray, Questionnaires including GERD-HRQL [24] and foregut symptom [25] as well as 24-h pH monitoring were performed at Baseline and at 6 months after surgery. The GERD-HRQL score is used as a screening tool at the 1-year follow-up to determine whether further investigations are necessary, including: additional contrast swallow x-ray and 24-h pH monitoring as well as gastroscopy and manometry (Fig. 2).

An independent Data Monitoring Committee (DMC) evaluated the safety data and selected efficacy data. The DMC could have recommended increasing the sample size if they found that this was motivated.

Primary safety objective was to assess the incidence of serious adverse device effects (SADEs) and procedure-related serious adverse events (SAEs). Primary efficacy objective was to assess the percent reduction from baseline of GERD symptoms based on the GERD-HRQL total score.

Secondary safety objective was to assess the incidence of adverse device effects (ADEs) and procedure-related adverse events (AEs). Secondary efficacy objectives were to assess the reduction or normalization from baseline of the total acid (pH < 4) exposure time on 24-h pH monitoring, to assess the reduction from baseline in the proportion of subjects using proton pump inhibitors (PPI) during the study, to assess the reduction from baseline of foregut questionnaire scores after the procedure and to assess the reduction from baseline of individual GERD symptoms based on the GERD-HRQL score.

Key study inclusion criteria were:

1. Subject’s age ≥ 18 years and ≤ 75 years;
2. Subject has documented typical GERD symptoms present for > 6 months which respond to PPIs as anti-GERD medication. Typical symptom of GERD is defined as heartburn, which is a burning epigastric or substernal pain;
3. Subject requires daily PPI anti GERD medication;
4. Subject has a 24-h pH monitoring proven GERD performed while off any anti-reflux medication or after discontinuation for at least 7 days prior to testing. Total distal esophageal pH must be ≤4 for ≥4.5% of the time during a 24-h monitoring.

Key study exclusion criteria were:

1. Subject has a history of gastroesophageal surgery, anti-reflux or bariatric procedure;
2. Presence of a para-esophageal hernia or sliding hernia of > 3 cm determined on endoscopy;
3. Presence of esophageal dysmotility disorder such as but not limited to scleroderma, achalasia, Nutcracker esophagus;
4. Presence of an esophagitis grade C or D according to the Los Angeles classification;
5. Subject has a body mass index (BMI) > 35 kg/m^2^;

The study was carried out in accordance with the Declaration of Helsinki, and the Regional Ethics Committee approved the study protocol.

### Study statistical analysis

#### Statistical methods

A sample size calculation was performed for this study where 45 subjects (50 including the drop-out rate) were determined to be sufficient. Continuous data is summarized using descriptive statistics and categorical data is presented using absolute frequency and percentage. The following analysis sets were considered in the statistical analysis:

- The full analysis set (FAS): in accordance with the intention-to-treat (ITT) principle, all subjects who received the device implant.
- The per-protocol (PP) analysis set: all subjects from FAS without any major protocol violations.
- The safety analysis set: all subjects who received the device implant.
- The primary efficacy variable was analyzed both in the FAS and in the PP analysis set. The secondary efficacy variables were analyzed in the FAS only. When timely results as per protocol were not available, subsequent results were used.

For all other variables, the safety analysis set was used.

#### Safety assessments

All AE summaries described below are presented for each of the following groups of AEs: All reported AEs, ADEs, AEs related to surgery and AEs not defined as ADEs or surgery related AEs (i.e. not covered in 2 and 3 above). Summaries include: the number and percentage of subjects who reported at least 1 AE and the number of events reported by seriousness, severity and outcome, and by system organ class (SOC) and preferred terms (PTs). Also, number of AEs leading to death were summarized.

Procedure-related complications (intra-operative complications or device related implanting difficulties, peri-operative bleeding [> 500 mL], peri-operative perforation, wound infection, pulmonary complications), length of the subjects’ hospital stay, and weight are summarized descriptively. In addition, weight is also summarized as change from baseline.

#### Efficacy assessments

For the primary efficacy endpoint, the percent reduction from baseline of GERD symptoms based on the GERD-HRQL total score (questions 1–10) is summarized using descriptive statistics, including 95% CI of the mean percent reduction. Number of subjects with a worsening from baseline is summarized. Total score values for each subject and visit are also presented. The data is also presented as the number of subjects obtaining at least a 50% improvement of the baseline figures. The aim of this analysis was to show that the lower limit of the CI exceeded 60%. Comparisons between values at baseline versus post-procedure follow-up were performed using a paired t-test at the 0.5 significance level. Month 6 is considered the primary efficacy endpoint. All secondary efficacy endpoints are presented descriptively, as appropriate.

#### RefuxStop™ operating procedure

All steps to treat hiatal hernia (HH), if present, should be followed:

1. Hernia repair consisting of complete reduction of HH, and sac excision if present
2. Extensive mediastinal esophageal dissection with vagal preservation
3. Gastroesophageal junction fat pad dissection to expose the angle of His
4. Intraoperative evaluation of esophageal length (with no traction)
5. About 1 cm additional dissection with small 1 cm down traction on esophagus
6. Tension-free crural repair
7. Left side adhering fundus to esophagus all the way up to the diaphragm (using the above mentioned small 1 cm down traction on esophagus)
8. GERD management with RefluxStop™ as an anti-reflux procedure including placing RefluxStop™ in a pouch outside the top part of the fundus close to esophagus

Few issues need to be emphasized. For the new treatment principle to work the device needs to be placed high-up, clearly above the upper edge of the LES. To achieve such result; firstly, the dissection around esophagus in mediastinum needs to be more extensive and as high up as possible. Secondly, the adherence of the stomach fundus wall to the esophagus, which builds the platform for the device, should also be placed as high up as possible and include the esophagus on the subjects left side only.

**Fig. 3:**
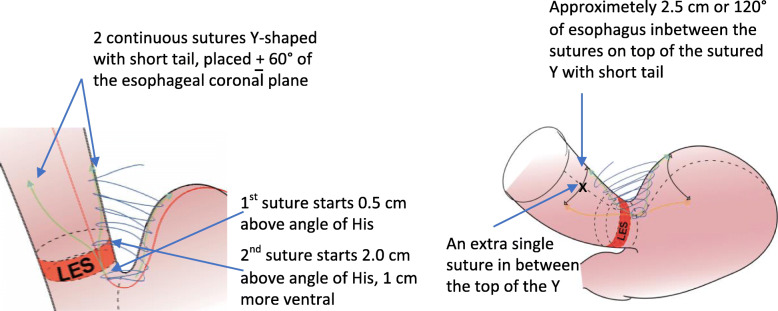
Simplified stomach to esophagus Y-shaped suturing for smaller sized fundus

The left lateral part of the esophagus should be attached to the stomach fundus wall. If the fundus is on the larger side, three parallel continuous non-resorbable sutures could be used, and when fundus is on the smaller side, two continuous sutures could be sutured in a Y-shape with short tail with one additional single suture in between the top of the Y (Fig. 3). The latter single suture could be replaced if sutures with hooks are used (for example non-resorbable V-Loc) with the top of the Y-shaped sutures angling slightly downwards and inwards towards each other to further stabilize both the sutures and the top part of the adherence between the fundus and the esophagus. We advocate one should avoid fat in the suture line attachments as much as possible and avoid suturing with too superficial sutures in the esophagus.

**Fig. 4:**
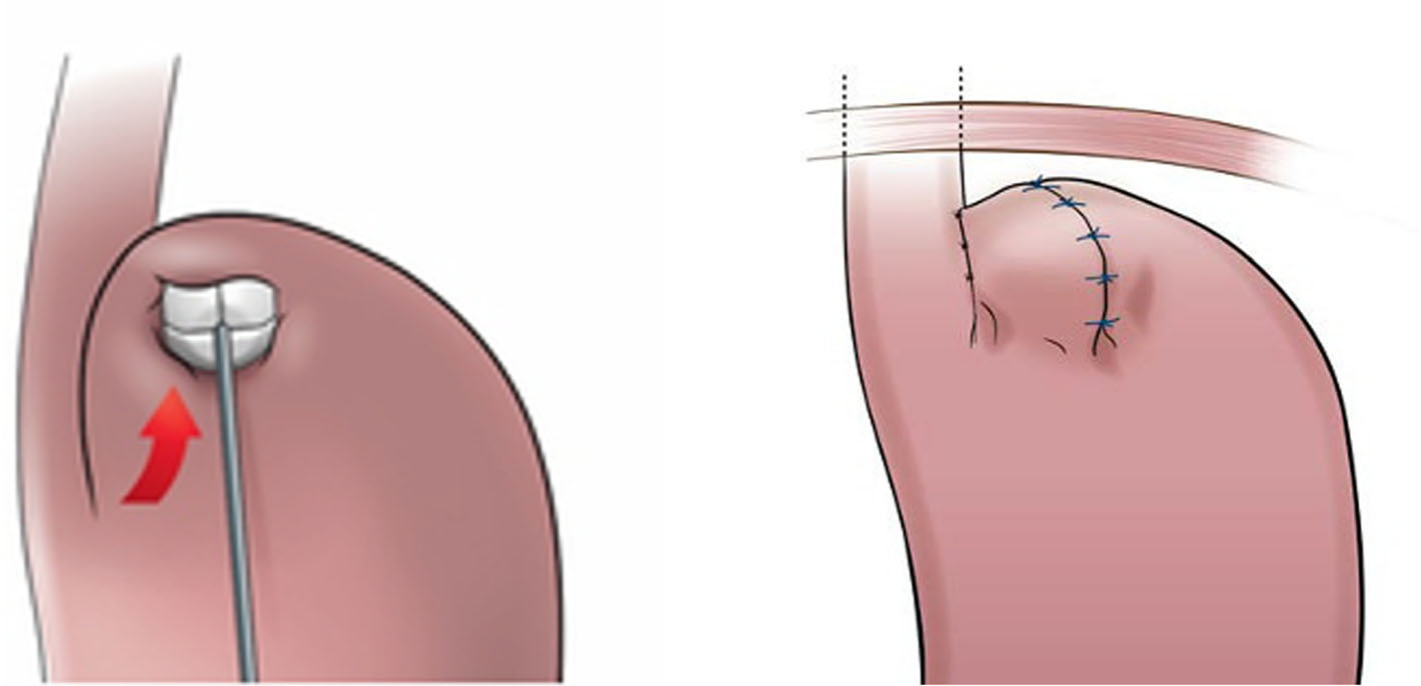
The device is positioned using the RefluxStop™ deployment tool

The placement of the device is performed using a special instrument that also compresses the device before introducing it. The device is then placed high-up on the outside of the stomach fundus wall and invaginated/covered by stomach tissue in a pouch to keep it in place, performed when holding the device with the instrument to ensure its high-up placement (Fig. 4).

**Fig. 5:**
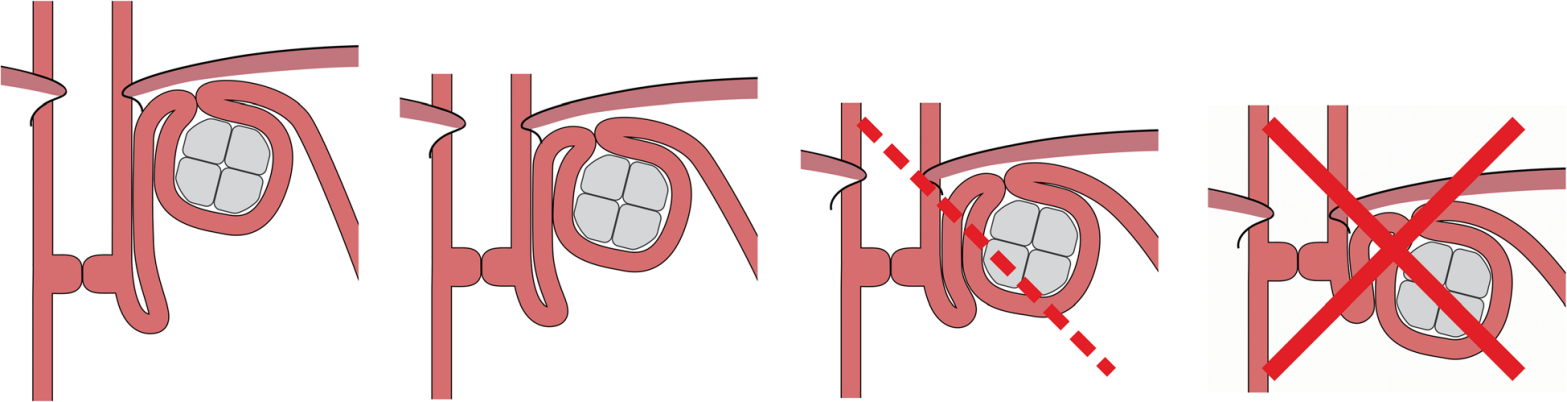
RefluxStop™ Height Categories. The categories of the device placement with a high-up placement important for a successful result. Optimal (1), top of the device placed > 1 time the device size above the upper edge of the LES, Acceptable (2), top of the device placed 0.5–1 time the device size above the upper edge of the LES, and Failure Risk (3), top of the device placed 0–0.5 time the device size above the upper edge of the LES, which has a high risk of failure in the mid- to long-term, and in addition Unacceptable (4), when the position of the RefluxStop™ device is fully below the upper edge of the LES in which case the device cannot function properly, (likely leading to failure immediately or in the short- to mid-term)

Due to the importance of positioning the device high-up and to ensure quality control of the procedure, the device shall be categorized using the following scale: **1 Optimal**, top of the device placed > 1 time the device size above the upper edge of the LES; **2 Acceptable**, top of the device placed 0.5–1 time the device size above the upper edge of the LES; and **3 Failure Risk**, top of the device placed 0–0.5 time the device size above the upper edge of the LES, which has a high risk of failure in the mid- to long-term; and in addition **4 Unacceptable**, when the position of the RefluxStop™ device is fully below the upper edge of the LES, in which case the device cannot function properly (likely leading to failure immediately or in the short- to mid-term) (Fig. 5).

It is also of utmost importance to place the device close to the esophagus for the device to function properly. Therefore, each height category should also be classified by **Close** (C), a placement of < 1.5 cm from the esophagus where primarily just the thickness of the doubled stomach wall is placed between the device and the esophagus, or **Distant** (D), a placement further than 1.5 cm away from the esophagus (Fig. 5).

## Results

### Study subjects

The 50 subjects had the procedure performed at four different sites and have been followed for 1 year. Device implantation was attempted and performed in all subjects. Three subjects had discontinued the study by 1 year:

- 2 subjects with successful treatment results discontinued the study at 3 months and 6 months, respectively, with the following results at the time of discontinuation:
  - No Dysphagia, no Odynophagia, no Regular Daily PPI consumption, no Regurgitation and subjects Satisfied
- 1 subject had the device placed too low at surgery – refused re-surgery and discontinued at 6-months with the following results at the time of discontinuation:
  - Moderate Dysphagia, no Odynophagia, no Regular Daily PPI consumption, minimal Regurgitation and subject Dissatisfied

### Baseline characteristics

Subject mean age was: 51.5 (SD 11.8) years. Of the 50 subjects, 28 (56%) were men and 22 (44%) women. PPI consumption was halted for all subjects at least 1 week prior to the baseline visit. 24-h pH monitoring (percentage of overall time pH < 4) at baseline showed acid reflux in all subjects; mean result 16.35%. The mean score for the GERD-HRQL questionnaire (questions 1–10) 1 week off PPI at baseline was 28.8 (SD 7.3). Before surgery, all subjects took PPIs. At baseline endoscopy, 13 subjects (26.0%) had Grade A esophagitis and 9 subjects (18.0%) had Grade B esophagitis. At baseline, 26 subjects reported severe regurgitation in the foregut questionnaire. Forty-five of 50 subjects were Dissatisfied, 4 subjects Neutral and 1 subject Satisfied at baseline. The median score for severity of heartburn (GERD-HRQL question 1) was 4.0 at baseline (range 0 to 5). When assessed by the GERD-HRQL questionnaire at baseline, 15 subjects had difficulty swallowing (dysphagia) and 13 subjects had pain at swallowing (odynophagia). The median score for bloating or a gassy feeling was 4.0 (range 0 to 5) at baseline.

### Safety parameters

No serious adverse events related to the RefluxStop™ device were reported during the 6-month and 1-year follow-up periods. No deaths, no device deficiencies and no device explantations were reported/performed during this period.

The following presentation of the adverse events has been divided into events related to the device, anti-reflux surgery or surgical procedure in general (Table 1). Further division of events with respect to seriousness aims to facilitate an overview of the safety profile of the device.

**Table 1:** Adverse Events related to the device, anti-reflux surgery or surgery in general

Adverse Events during surgery and in the postoperative course up to 6 months	Number of subjects n (%)	Number of events n	Full recovery with intact treatment effect
Number of Adverse Events (*n* = 50)	8 (16%)	11	YES
Device related adverse events (SADE or ADE)	0	0	
Surgical non-device related serious adverse events (SAE)	4 (8%)	6	YES
Severe			
Mediastinal abscess, empyema and abdominal abscess	1^a^	3	YES
Intra-abdominal haemorrhage	1	1	YES
Moderate			
Pleuritis	1	1	YES
Mild			
Removal of foreign body (part of a needle from the abdominal wall)	1	1	YES
Surgical non-device related non-serious adverse events (AE)	4 (8%)	5	YES
Moderate			
Abdominal pain and incisional hernia	1^b^	2	YES
Mild			
Accidental intra-operative instrumental hepatic lesion (small)	1	1	YES
Post-op delayed gastro-intestinal paralysis (one day)	1	1	YES
Procedural pneumothorax	1	1	YES
**Adverse Events between 6 months and 1 year**^**c**^	**1 (2%)**	**1**	**YES**
Device related adverse events (SADE or ADE)	0	0	
Surgical non-device related serious adverse events (SAE)	1 (2%)	1	YES
Moderate			
Release of fundoplication sutures – successfully re-sutured	1	1	YES

The analysis is based on 50 subjects, i.e. all subjects included in the safety analysis set

^a^ The events occurred in the same subject (caused by an infection unrelated to the device, which was unaffected in its enclosed pouch)

^b^ The events occurred in the same subject (a small hernia in the abdominal wall)

^c^ Four AEs of gastritis occurred in 4 subjects all with endoscopy verified gastritis and lack of esophagitis, all resolved

#### Serious adverse events related to surgery – none device related

Six serious adverse events (SAEs) were reported for 4 subjects at 6 months. All but one of the events described above were resolved at the time of the 6-months analysis cut-off date. The subject diagnosed with an abdominal wall hernia unrelated to surgery may eventually undergo surgical repair.

Two of these adverse events above were more serious, directly correlated to surgery in general belonging to the two most common adverse events in surgery, namely one infection and one bleeding. The bleeding lead to a second look. It probably originated from the divided short gastric vessel, was self-limiting and clot evacuation and drainage was performed. The infection included both mediastinal abscess and empyema, probably due to the infected mediastinal haematoma. However, due to a well-performed invagination of the device the infection did not spread to the pouch with the device, and the subject healed completely with an excellent treatment result.

One serious adverse event (SAE) was reported in 1 subject between the 6-month and 1-year visit due to the release of fundoplication sutures. Reoperation was performed, and the subject had a successful 1-year follow-up visit. This confirms the treatment principle and reinforces the importance of positioning the device high up.

#### Complications and side effects related to the procedure

##### Dysphagia and odynophagia

Two important complications related to anti-reflux surgery are dysphagia and odynophagia [1–3, 20]. Fifteen subjects had dysphagia at baseline, whereof in 11 completely resolved and 4 subjects continued to have reduced swallowing problems at the 6-month visit (GERD-HRQL score above 1) (Table 2). At the 1-year visit, 2 subjects reported minimal dysphagia. No new cases of dysphagia were recorded at either visit.

**Table 2:** Dysphagia and Odynophagia at Baseline, 6-months and 1-year post-operation

**Dysphagia**	**Baseline (n = 50)**		**6-months (n = 47)**		**6-m** ***p*-value**	**1-year (n = 47)**		**1-yr** ***p*-value**
None 0–1	35	70%	43	91%		45	96%	
Minimal 2	4	30%	3	9%	< 0.001	2	4%	< 0.001
Moderate 3	6		1			0		
Severe 4–5	5		0			0		
**Odynophagia**	**Baseline (n = 50)**		**6-months (*n*** **= 47**)		**6-m** ***p*-value**	**1-year (n = 47)**		**1-yr** ***p*-value**
None 0–1	37	74%	47	100%		46	98%	
Minimal 2	5	26%	0	0%	< 0.001	1	2%	
Moderate 3	3		0			0		< 0.001
Severe 4–5	5		0			0		

Thirteen subjects had pain at swallowing before surgery and none of the subjects reported pain at swallowing at the 6-month visit, while at the 1-year visit, one subject reported pain at swallowing at the same minimal level as at baseline. No new cases of odynophagia were recorded at the 6-month or 1-year visits (Table 2).

### Efficacy parameters

#### Reduction from baseline of GERD symptoms based on the GERD-HRQL score

**Fig. 6:**
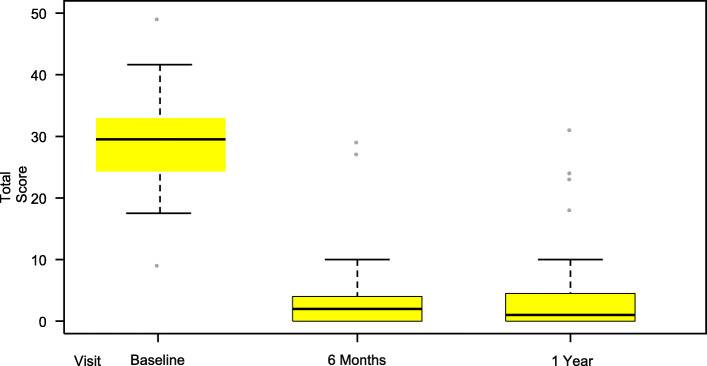
Total GERD-HRQL score at Baseline, 6 months and 1 year post-operation. Total GERD-HRQL score including all 10 items before and 6 months after RefluxStop™ surgery, showing significant change from baseline to 6 months and 1-year (*p* < 0.001). Three subjects had less than 50% improvement of the score at 1-year, whereof two subjects due to reasons other than GERD and 1 subject due to a too low positioning of the device thereby prohibiting its function

The mean total GERD-HRQL score at baseline was 28.8 (SD 7.3), *n* = 50. At 6 months (*n* = 47), the score had decreased to 3.4 (SD 6.0) (*p* < 0.001), reflecting an improvement in GERD symptoms of 88%. One subject discontinued the study with successful 3-month score (Fig. 6 and Table 3).

**Table 3:** GERD-HRQL total score at 6 months and 1 year post-operation

Full analysis set	Percent change Baseline to 6 M	Percent change Baseline to 1 Y
n	47	47
Mean (SD)	−89.0 (17.9)	−86.2^a^ (24.5)
Median	−94.6	−95.2
Min, Max	−100.0, −15.6	−100.0, 9.1
*p*-value	< 0.001	< 0.001
95% CI	(−94.3, −83.7%)	(−93.4, −79.0%)

^a^Includes 2 subjects with < 50% improvement for reasons other than GERD and one with the device positioned improperly in a too low position

Forty-five out of 47 subjects had at least 50% improvement of the GERD-HRQL total score from baseline. Two subjects are defined as a failure not having a 50% improvement in the GERD-HRQL score. Both these subjects had the device positioned too low, prohibiting the device to function as intended. One subject had the device placed too low at surgery and refused re-surgery (representing proof of concept since an incorrectly placed device does not treat the subject). In the second subject, the suture line between the stomach and esophagus failed which is believed to be due to fat in the suture line attachment. The subject was well treated during the 3-month follow-up and was recorded as a failure after 6 months because the fundus with the intact invaginated device had slipped down due to the stomach-to-fundus suture line failing, resulting in the device being positioned too low, hindering its function. The subject was reoperated after 8 months with resuturing and was immediately treated with optimal results (GERD-HRQL total score zero points) supporting once again that the new treatment principle works.

At the 1-year follow-up visit (*n* = 47), > 50% improvement in GERD symptoms compared to baseline was reported in 44 subjects. Two subjects with < 50% improvement were shown to not have GERD and one subject had the device positioned improperly too low. The average score improvement when deducting the two subjects with failed results for reasons other than GERD was 89% and average score was 3.2. Full results in Fig. 6 and Table 3.

#### 24-h pH monitoring

**Fig. 7:**
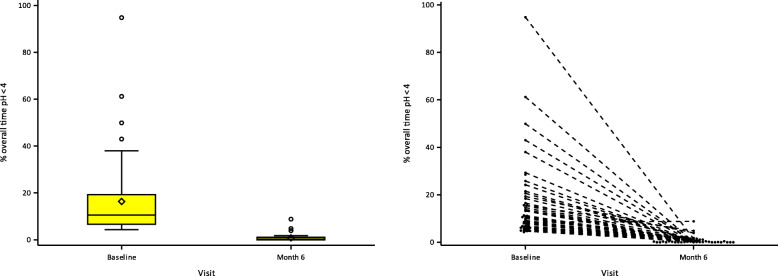
24-h pH monitoring at Baseline and 6 months post-operation. 24-h pH monitoring mean reduction from baseline of 16.35 to 0.80% at 6-month visit. Normal 24-h pH results in 98% of subjects

pH testing at the 6 months study visit was completed by 45 subjects. The esophageal pH was monitored using Bravo Capsules during a 24-h period at baseline and again at 6 months post-implantation (Fig. 7 and Table 4). The results show a mean reduction from baseline of percentage of overall time with pH < 4 from 16.35 to 0.80% at the 6-month visit (*p* < 0.001) reflecting a 95% improvement of the mean value. Normal 24-h pH results in 98% of subjects.

**Table 4:** 24-h pH monitoring at Baseline and 6 months post-operation

% overall time pH < 4	Baseline	6 Months	Change Baseline to 6 Months
n	50	45	45
Mean (SD)	16.35% (16.60)	0.80% (1.56)	−16.0 (17.46)
Median	10.55%	0.30%	−10.05
Min, Max *p*-value*	4.3, 94.8	0.0, 8.8	−93.4, 0.3 (*p* < 0.001)

* Paired t-test of the mean change

Two of the subjects refused the burdensome pH-monitoring test whereof one had a successful GERD-HRQL questionnaire score and one subject had a to too low position of the device already at surgery, prohibiting the function of the device. This later subject refused the pH monitoring test and re-surgery

Two of the subjects refused the burdensome pH-monitoring test whereof one had a successful GERD-HRQL questionnaire score and one subject had the device positioned too low at surgery, prohibiting the function of the device. The latter subject refused reoperation and the pH monitoring test, but notable that this subject did not take any PPIs.

#### PPI medication

PPI consumption/reuse is often used as an indicator for success in reflux surgery. Before surgery all 50 subjects were taking PPI drugs. Six months after implantation no subject (0%), *n* = 47, took PPI medication. At the 1-year follow-up visit, 1 subject (2.1%), n = 47, who had the device positioned too low, took regular daily PPIs (Table 5).

**Table 5:** PPI consumption before surgery and at follow up visits

	Number of subjects (n)	Total n (%)	PPI use related to acid reflux	PPI use related to malfunction of the treatment principle of RefluxStop™
Regular daily PPI consumption				
Baseline	50	50 (100%)	50 (100%)	50 (100%)
6 Months	47	0 (0.0%)	0	0
1 year	47	1 (2.1%)	1^a^	0

^a^One (1) subject with the device positioned too low, prohibiting the function of the device

#### Gas bloating

Gas bloating often occurs in anti-reflux surgery [1–3, 17] and in the investigation the question about gas bloating in the GERD-HRQL (score above 2) was present in 84.0% of the subjects at baseline and in 19.1% of the subjects at 1 year. Gas bloating at 1 year compared to baseline: disappeared in 30 subjects; improved in 7 subjects; remained unchanged in 2 subjects; and no subject had their gas bloating symptoms worsen (Table 6).

**Table 6:** Gas bloating including change between Baseline and 1-year

Gas bloating	Baseline (n = 50)		6-months (n = 47)		6-m *p*-value	1-year (n = 47)		1-yr *p*-value	Gas bloating change between Baseline and 1-year (subjects)
None 0–2	8	16%	37	79%		38	81%		Disappeared in 30
Minimal 3	10	84%	5	21%	< 0.0001	5	19%	< 0.0001	Improved in 7
Moderate 4	18		3			3			Unchanged in 2
Severe 5	14		2			0			Worsened in 0

#### Regurgitation

Daily regurgitation is common in acid reflux subjects [3] and occurred in 44 subjects (88%) at baseline in the investigation, whereof 33 subjects had moderate to severe regurgitation. At 1-year follow-up 46 out of 47 evaluable subjects (97.8%) operated with RefluxStop™ had none or minimal occasional episodes of regurgitation (Table 7).

**Table 7:** Daily regurgitation before surgery and at 1-year follow up visit

Daily regular Regurgitation	Baseline (n = 50)		1-year (n = 47)		1-yr *p*-value
None < 1/day	6	12%	43	91%	
Minimal 1–2/day	11	88%	3	9%	< 0.0001
Moderate 3–4/day	16		1		
Severe 5 or more per day	17		0		

#### Subject satisfaction

Forty-five subjects at baseline indicated that they were dissatisfied with their present condition whereas at follow-up 6 months after implantation 44 subjects (93.6%) were satisfied, one subject was neutral (2.1%) and two subjects dissatisfied (4.3%) (*n* = 47) (Table 8). The two dissatisfied subjects were the same subjects as discussed previously with the device placed too low, hindering its function. Out of these, one subject was reoperated between 6 months and 1 year to improve the positioning of the device by placing it higher up and was thereafter immediately satisfied again (HRQL total score zero).

**Table 8:** Subject satisfaction before surgery and at follow-up visits

Subject Satisfaction	Baseline (n = 50)	6-months (n = 47)	1-year (n = 47)
Satisfied	1	44	43
Neutral	4	1	1
Dissatisfied	45	2	1
Dissatisfied – not due to GERD	0	0	2^a^

^a^ Two of the dissatisfied subjects were dissatisfied for reasons other than GERD:

- One (1) subject had normal gastroscopy, 24-h pH monitoring and contrast swallow x-ray; and

- One (1) subject had gastritis and regained satisfaction after a short-term non-PPI treatment, still satisfied at the 2-year follow-up

At the 1-year follow-up, one subject was still neutral (2.1%) and one subject was dissatisfied due to the device being positioned too low. In addition, two subjects (4.3%) were dissatisfied for reasons other than GERD: one subject had all tests performed with a normal outcome and one (1) subject had short-term gastritis and was satisfied at the subsequent follow-up visit and 2-year visit.

So far, no correctly operated subject has failed, which means that there is a 100% success rate for the treatment principle of RefluxStop™, supporting a possible shift in acid reflux treatment.

### Summary of the safety endpoint

No device related serious or non-serious adverse events occurred during the study. The surgery related serious adverse events were all resolved with satisfactory results.

### Summary of the main efficacy endpoint

GERD-HRQL

- 86% score reduction from baseline at 1-year follow up visit
- 95% confidence interval: 94.1–79.4%
- Lower limit of confidence interval was above 60%
- Percent of subjects with at least 50% improvement: 93.6%
- 3 subjects had < 50% improvement, whereof:
  - 2 were shown not to have GERD
  - 1 due to too low position of the device thereby prohibiting its function

24-h pH Monitoring:

- 98% of subjects had normal 24-h pH monitoring (percentage of total time) at 6-months
- 95% improvement at 6-months on mean value
- 16.35% at Baseline reduced to 0.80% at 6-months (*p* < 0.001)

## Discussion

The new device RefluxStop™ uses a new dynamic acid reflux treatment principle, which does not compress the food passageway. The 1-year results indicate that the new principle for treating acid reflux is successful for all correctly operated subjects.

That a dynamic treatment for acid reflux, which avoids compressing the food passageway, would reduce complications such as dysphagia and odynophagia, as well as gas bloating, in relation to standard of care methods would be expected and is supported by the clinical trial results. No new diagnosed case of dysphagia was detected. In addition, dysphagia scores improved significantly at the 1-year follow-up visit compared to baseline. Furthermore, there were no device deficiencies and no complications related to the device itself.

Gas bloating is a common finding after fundoplication [26]. Interestingly, the prevalence of gas bloating is surprisingly reduced after RefluxStop™ treatment. One out of many reasons causing gas bloating could be inability to turn back swallowed air, either by belching or more likely due to unnoticeable air exchange over the LES. Avoiding compression of the esophagus allows swallowed air to be released up the esophagus thereby reducing gas bloating. Thus, these one-year results already indicate that this new dynamic acid reflux treatment principle is here to stay.

More surprisingly there are also indications that the treatment efficacy of the RefluxStop™ procedure may be an improvement over the current standard procedures. RefluxStop™ is effective to treat GERD symptoms and significantly reduces esophageal acid exposure. The GERD-HRQL questionnaire results show 86% score reduction from baseline at 1-year follow up visit and 89% score reduction when excluding the two subjects who had failed score result for reasons other than GERD. In addition, results obtained by 24-h pH monitoring showed 95% improvement at 6-months on mean value down to an average 0.8% of total time pH < 4. No other reason for treatment failure than inaccurate too low position of the device has been identified so far.

**Fig. 8:**
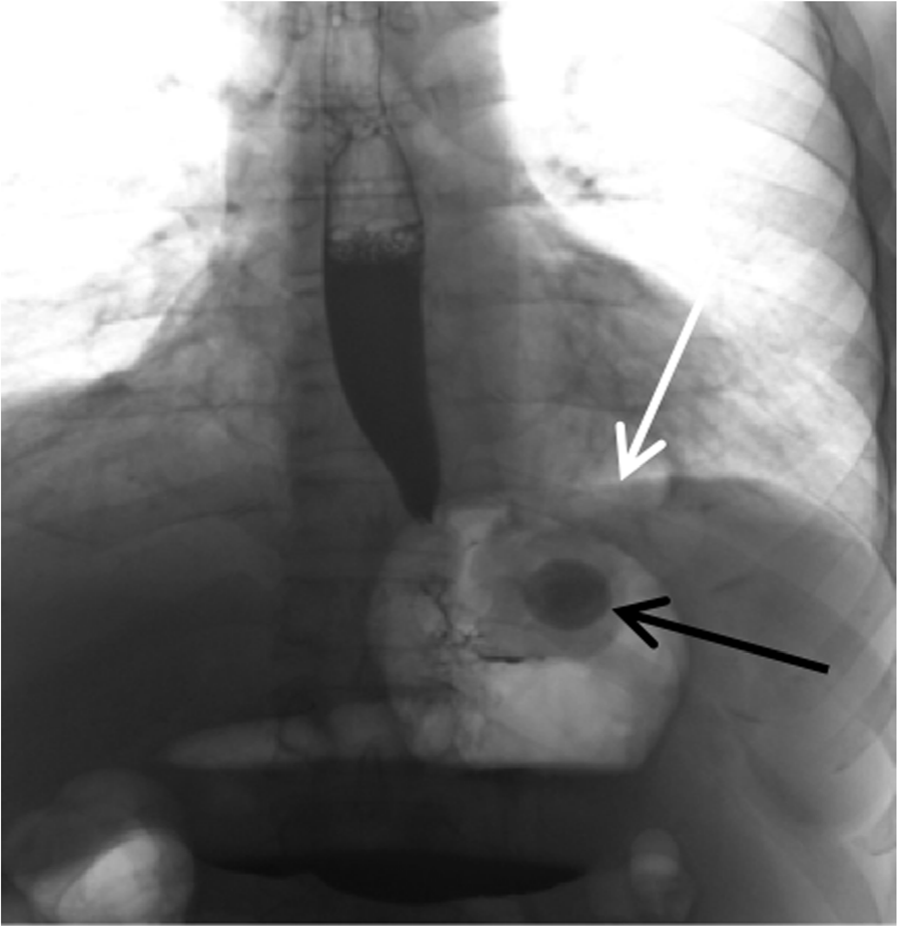
The RefluxStop™ device placed too low

**Fig. 9:**
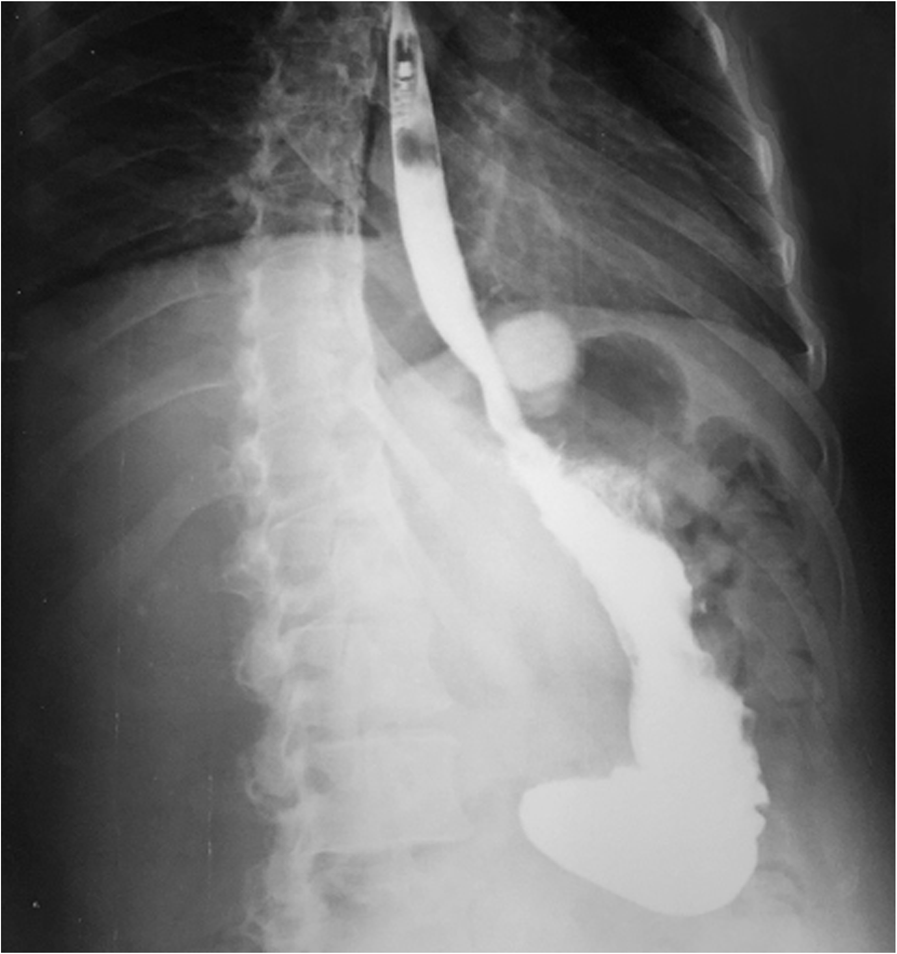
A correctly placed RefluxStop™ device

The new RefluxStop™ anti-reflux procedure has two important areas of consideration: **Firstly, the device only works if it is placed clearly higher up than the upper edge of the lower esophageal sphincter.** The platform for achieving such a result is to maximize the esophagus dissection all the way up in mediastinum (type B dissection) [27]. Then gastroesophageal junction fat pad dissection to expose the angle of His and an optimal reconstruction of the angle of His are mandatory, allowing a high-up placement of the device. Incorrect placement will result in treatment failure with no or limited reflux symptom relief, as suggested by the two subjects with the device placed too low, and correct placement of the device will result in a successfully treated subject. One subject had the device placed too low already at surgery, see two x-rays (Figs. 8 and 9). In addition, due to some expected adaption of tissue and degeneration of the thickness of the fundus top wall as a result of immobilization, the device positioning is a key factor for long-term success.

**The second important consideration is the adherence of the stomach fundus wall to esophagus** on the left lateral side, which builds the platform for the device and should be performed as high up as possible. The esophagus should be robustly attached to the stomach fundus wall. If the fundus is a bit larger in size, three parallel vertical continuous sutures could be used, and when the fundus is smaller, two continuous sutures could be sutured in a Y-shape with short tail and one additional single suture in between the top of the Y-shaped sutures. We advocate that one should avoid fat in the suture line attachments and avoid suturing with too superficial sutures in the esophagus.

The placement of the device with the specially designed instrument using compression of the device during introduction is a small part of the procedure. The instrument is used to hold the device in a high-up position when invaginated/covered completely by the stomach fundus wall.

Similar to existing anti-reflux surgery, the procedure as such can have complications of importance that require special care: bleeding and infection [28]. Infection could be a serious issue when placing an implant, however, the consequences of an infection are reduced if the device is completely covered/invaginated in the stomach wall by stomach-to-stomach sutures, thereby avoiding that the infection is spread to the device. Specific care is required to avoid gastric or esophagus injury/perforation. Because the esophagus is lacking a serosa that protects the muscle wall, sutures could cut through the muscular tissue if one is not careful enough.

We found the GERD-HRQL questionnaire to be a reliable screening tool to find subjects who need to perform 24-h pH monitoring. A failed questionnaire could occur for several reasons other than GERD, however, a successful questionnaire is very reliable and may only involve so-called silent acid reflux, subjects who are not included in the study from the beginning due to lack of symptoms. Most importantly, this group of subjects who have a failed questionnaire (symptoms of acid reflux) is more motivated to perform the often burdensome 24-h pH monitoring.

A larger PMCF survey is currently being set up to further evaluate subjects long-term. The results so far indicate that RefluxStop™ may become a breakthrough in acid reflux treatment.

## Conclusion

The study results confirm that RefluxStop™ is safe and well tolerated with no complications related to the RefluxStop™ device itself (no SADE or ADE). Overall, data from the present study show that the complications caused by the compression of the food passageway i.e. dysphagia, odynophagia and gas bloating are not an issue with the RefluxStop™ procedure. Both the GERD-HRQL score and 24-h pH monitoring results indicate success for the new treatment principle in all correctly operated subjects. These 1-year results are very promising and RefluxStop™ may cause a shift in acid reflux treatment, although further studies and follow-up are needed.

## Data Availability

The datasets used and/or analyzed during the current study are available from the corresponding author on reasonable request.

## Abbreviations

GERD: Gastroesophageal reflux disease
HRQL: Health Related Quality of Life Questionnaire
CE-Mark: Conformité Européenne, a European certification mark
LES: Lower esophageal sphincter
PPI: Proton Pump Inhibitor
FAS: Full analysis set
PP: Per-protocol
ITT: Intention-to-treat
AE: Adverse events
SAE: Serious adverse events
SADE: Serious adverse device effects
ADE: Adverse device effects
DMC: Data Monitoring Committee
SOC: System organ class
PT: Preferred terms
CI: Confidence interval
HH: Hiatal hernia
PMCF: Post-market clinical follow-up
